# Novel long-range regulatory mechanisms controlling *PKD2* gene expression

**DOI:** 10.1186/s12864-018-4892-6

**Published:** 2018-07-03

**Authors:** Stéphanie Moisan, Stéphanie Levon, Emilie Cornec-Le Gall, Yannick Le Meur, Marie-Pierre Audrézet, Josée Dostie, Claude Férec

**Affiliations:** 1 Institut National de la Santé et de la Recherche Médicale (INSERM), U1078, Brest, Bretagne France; 2 Faculté de Médecine et des Sciences de la Santé, Université de Bretagne Occidentale (UBO), Brest, Bretagne France; 3 Laboratoire de Génétique Moléculaire et d’Histocompatibilité, Centre Hospitalier Régional Universitaire (CHRU), Hôpital Morvan, Brest, Bretagne France; 4 Service de néphrologie, Centre Hospitalier Régional Universitaire (CHRU), Brest, Bretagne France; 5 Department of Biochemistry and Goodman Cancer Research Center, McGill University, Montréal, Québec Canada; 6 Etablissement Français du sang (EFS), Brest, Bretagne France

**Keywords:** ADPKD, *PKD2*, Chromatin organization, Enhancer, Gene expression, Transcription regulation

## Abstract

**Background:**

*Cis*-regulatory elements control gene expression over large distances through the formation of chromatin loops, which allow contact between enhancers and gene promoters. Alterations in *cis*-acting regulatory systems could be linked to human genetic diseases. Here, we analyse the spatial organization of a large region spanning the polycystic kidney disease 2 (*PKD2*) gene, one of the genes responsible of autosomal dominant polycystic kidney disease (ADPKD).

**Results:**

By using chromosome conformation capture carbon copy (5C) technology in primary human renal cyst epithelial cells, we identify novel contacts of the *PKD2* promoter with chromatin regions, which display characteristics of regulatory elements. In parallel, by using functional analysis with a reporter assay, we demonstrate that three DNAse I hypersensitive sites regions are involved in the regulation of *PKD2* gene expression.

**Conclusions:**

Finally, through alignment of CCCTC-binding factor (CTCF) sites, we suggest that these novel enhancer elements are brought to the *PKD2* promoter by chromatin looping via the recruitment of CTCF.

**Electronic supplementary material:**

The online version of this article (10.1186/s12864-018-4892-6) contains supplementary material, which is available to authorized users.

## Background

Autosomal dominant polycystic kidney disease (ADPKD) is one of the most common Mendelian genetic disorders with an incidence of 1/400 to 1/2000 affected individuals worldwide [1]. The disorder is heterogeneous with two main genes, polycystic kidney disease 1 (*PKD1*) and 2 (*PKD2*), which are responsible for most disease cases [2, 3]. While *PKD1* accounts for 75–80% of patients, 10–15% of patients have defects in *PKD2*. A third gene, *GANAB* has recently been identified as accounting for less than 1% of affected patients [4]. Although more than 1200 *PKD1* and almost 200 *PKD2* pathogenic mutations have been reported (ADPKD Mutation Database, http://pkdb.mayo.edu/), a significant proportion of patients with ADPKD symptoms (5–10%) do not appear to carry these pathogenic gene mutations [5–7]. There is significant clinical heterogeneity in the severity of the disease among patients as well as within the same family.

*PKD1* and *PKD2* encode two transmembrane glycoproteins, namely polycystin-1 (PC1) and − 2 (PC2) which interact via their C-terminal tails [8]. The regulated expression of the PC1/PC2 complex is crucial to understand cyst formation and eventually modulate cyst expression.

The correct expression of a gene relies on the regulatory mechanisms that control it. A part of the regulatory network consists of *cis*-regulatory sequences, such as enhancers and insulators, which may be located far away from their target gene [9–12]. These regulatory elements control gene expression over long distances via the formation of chromatin loops allowing physical proximity between enhancers and gene promoters [13]. Remote regulatory sequences can be identified by mapping DNAse I hypersensitive sites (DHS), which correspond to regions where DNA is more accessible [14–16]. In this context, variations in chromatin organization or alterations of long-range acting elements may impact gene expression. Mutations of these *cis*-acting regulatory sequences can result in human genetic diseases [17, 18], such as aniridia [19] or blepharophimosis syndrome [20, 21]. Therefore, to fully decipher gene expression and genetic disorders, it is particularly important to investigate genetic regulatory networks.

Since the regulation of polycystin is crucial to understand cystogenesis, we decided to explore the long distance *cis*-regulation of *PKD2*. The *PKD2* gene is expressed in several tissues and more particularly in the kidney. In addition to its tissue-specific expression, *PKD2* is also temporally regulated [22]. Moreover, it has been shown that the PC levels can be strictly controlled [23–26]. The mechanisms controlling the tight regulation of *PKD2* expression remain poorly characterized and understood. The *PKD2* promoter does not feature basic regulatory elements such as a TATA or CCAAT box, is GC-rich, and contains multiple putative Sp1, NF-1 and AP-2 protein binding sites [27, 28]. Computational analyses and experimental assays have also described other putative transcription factor binding sites in the *PKD2* promoter region [29–31]. Moreover, several studies have shown that *PKD2* expression is regulated by numerous protein interactions including PKD1 or TRPC1 [32–38]. This control can also occur by other binding protein partners through 5′ and 3′ untranslated regions of *PKD2* mRNA [39–42].

Recent progress in molecular biology and bioinformatics analyses has allowed the development of new techniques to analyse the spatial organization of chromatin. The chromosome conformation capture (3C) technique developed by Dekker and colleagues in 2002 [43] measures the level of contacts between any two defined chromatin regions, and subsequent high-throughput approaches are used to map chromosomal interactions at the scale of the genome. Among these, the chromosome conformation capture carbon copy (5C) measures interaction frequencies within large chromosomal regions at high resolution [44]. We recently used this approach to identify several *CFTR* enhancers [45] and applied it here to a large region spanning the *PKD2* locus in primary human cyst renal epithelial cells. We choose to firstly conduct our long-range *cis*-regulation study with the *PKD2* gene because of challenging molecular analyses of the *PKD1* gene [46]. Approximately 70% of the *PKD1* gene (exons 1–33) is duplicated six times within six pseudogenes (*PKD1P1* to *PKD1P6*), which share a 97.7% sequence identity and a high GC content [47]. We identify novel contacts of the *PKD2* promoter with chromatin regions, which display characteristics of regulatory elements. In parallel, by using functional analysis with a reporter assay we demonstrate that three DHS regions are involved in the regulation of *PKD2* gene expression. Finally, through alignment of CCCTC-binding factor (CTCF) sites, we suggest that these novel enhancer elements are brought to the *PKD2* promoter by chromatin looping via CTCF.

## Results

### Analysing the human *PKD2* locus with the 5C technology

Very little is known about the regulatory elements that control *PKD2* transcription. To date, no study has examined the involvement of long-range regulatory elements in the mechanisms controlling *PKD2* expression. To pinpoint new regulatory elements involved in the control of *PKD2* expression, we decided to carry out 5C analysis in primary human cells. We used renal cyst epithelial cells (CRC) isolated from patients with ADPKD (carrying a *PKD1* mutation) from the Genkyst cohort who had undergone nephrectomy.

We chose to seek the *PKD2* promoter-interacting partners in a larger locus. To delineate the domain where the *PKD2* promoter interactions are more likely to occur, we used Hi-C data generated from a human lung carcinoma cell line A549 (one of cell lines with highest expression of *PKD2*) [48–50]. Hi-C is a molecular technique, which allows genome-wide quantification of chromosomal interactions in cell populations [51]. This method revealed that chromosomes are organized into topologically associating domains (TADs) corresponding to regions enriched in chromatin contacts [48, 52].

TADs represent folded DNA regions, ranging in size from several hundred kilobases to a few megabases, which is rather conserved across cell types and species [53–55]. TADs are delimited by boundaries, and loci located in different TADs typically interact less frequently than those within the same TAD, suggesting that boundaries act as physical insulators.

**Fig. 1:**
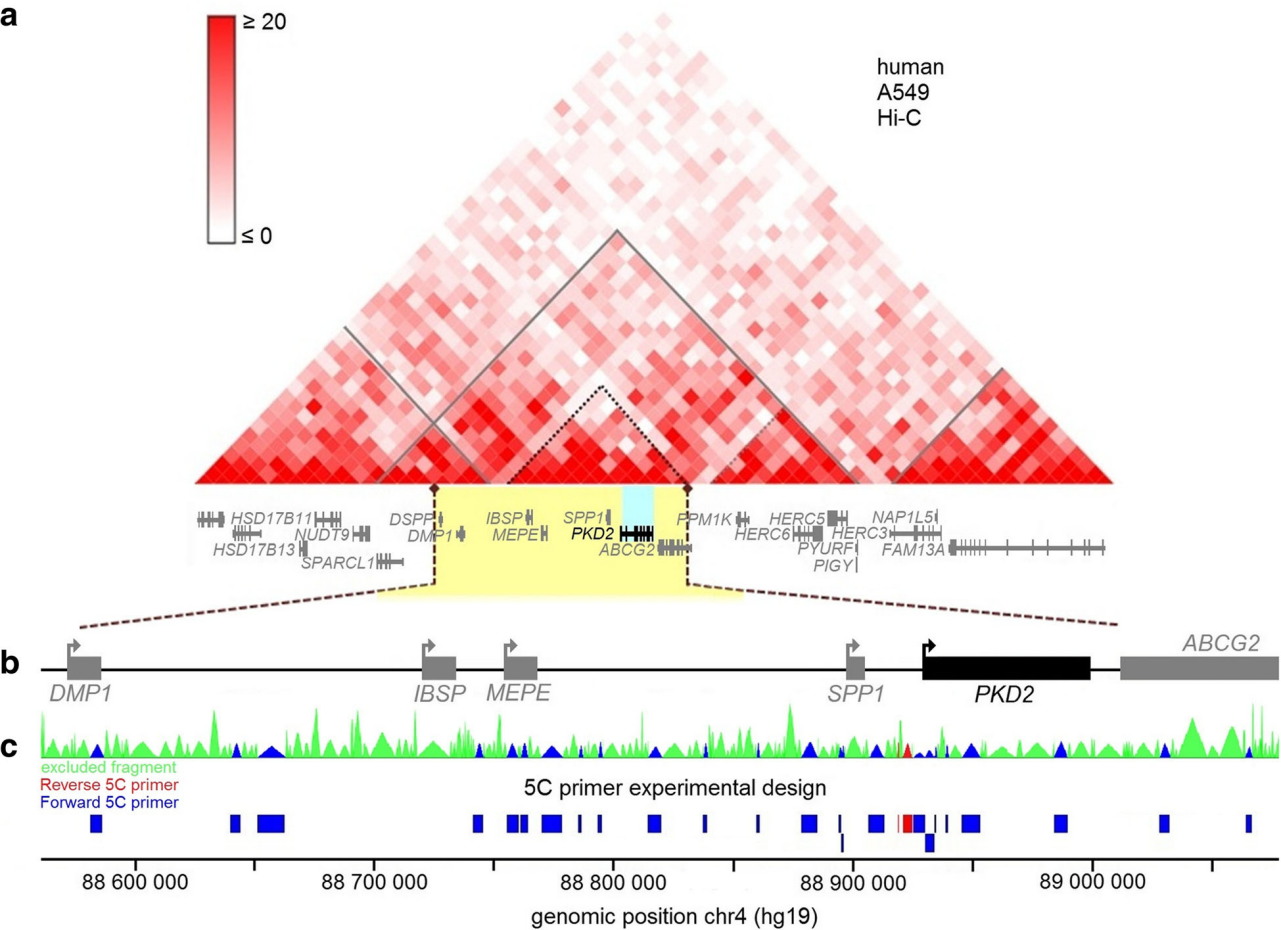
5C analysis of the human *PKD2* locus in primary human cells. **a** Hi-C data from the A549 human cell line reveal that *PKD2* lies in a large TAD that contains several other genes [48]. *PKD2* is further contained within a sub-TAD. The Hi-C data are shown in heatmap form where the increasing colour intensity reflects higher interaction frequencies between genomic regions in 40 kb-resolution bins. The grey lines outline the TADs while the dotted lines represent the sub-TADs. The genes contained within the marked area are shown under the heatmap. **b** Linear schematic representation of the ~ 540 kb genomic region characterized in this study. Arrows indicate transcription orientation. The *PKD2* gene is shown in black and the neighbouring genes in grey. **c** Anchored 5C primer design scheme used to map the genomic environment of the *PKD2* promoter. The two reverse “bait” 5C primers covering the *PKD2* promoter are shown in red. The 24 Forward 5C primers shown in blue represent the rest of the locus. The green areas indicate regions not probed by 5C. The region coverage is summarized at the top, and individual fragments are shown at the bottom. 5C primer sequences are found in Additional file 1: Table S1

TAD boundaries are enriched in CTCF sites [49], and analysis of the A549 Hi-C data revealed that *PKD2* lies within a TAD spreading from the *NUDT9* to the *PYURF* gene (Fig. 1a). *PKD2* itself localizes within a TAD substructure (sub-TAD) [56]. A second TAD overlaps with the domain containing *PKD2* and extends up to the *HSD17B11* gene. Because TADs are largely conserved between cell types, these data suggest that the chromosomal region between these genes may potentially interact with the *PKD2* promoter in different cell lines [48]. We therefore decided to focus our conformational analysis on an ~ 540 kb domain (hg19, chr4: 88,560,000-89,230,000) encompassing *PKD2* and its flanking genes: *DMP1, IBSP, MEPE, SPP1* and *ABCG2* (Fig. 1b). We used 5C-seq to detect and measure chromosomal regions interacting with the *PKD2* promoter in *cis* [44, 57]. The 5C technique has previously been used to detect chromatin organization and map physical contacts between promoters and regulatory elements [45, 56, 58, 59]. Similar to Hi-C, the 5C method quantifies interaction frequencies between DNA regions in fixed cells. The 5C protocol begins with the production of a 3C library where cells are fixed with formaldehyde to capture protein-protein and DNA-protein interactions. The fixed chromatin is then digested with a restriction enzyme followed by a ligation step that allows the production of pair-wise ligation products at frequencies corresponding to physical proximity between chromatin segments in vivo. The resulting 3C library is converted into a 5C library by a modified ligation-mediated amplification (LMA) procedure where Forward and Reverse 5C primers are annealed and ligated at 3C junctions in a multiplexed setting. 5C library is then amplified by PCR and processed for deep sequencing. We designed 5C primers within our study locus to measure interaction frequencies between the *PKD2* promoter (represented by 2 Reverse 5C primers or “baits”) and the rest of the ~ 540 kb domain (Fig. 1c; Additional file 1: Table S1). The 2 reverse primers selected as anchors in this work are corresponding to the most relevant EcoRI restriction fragments upstream to the *PKD2* TSS. Primers of restriction fragments closest to the TSS have been excluded because of quality parameters, interference and weak results. This 5C design therefore provides information about the genomic environment around the *PKD2* promoter in vivo.

### *PKD2* promoter is engaged in long-distance interactions

**Fig. 2:**
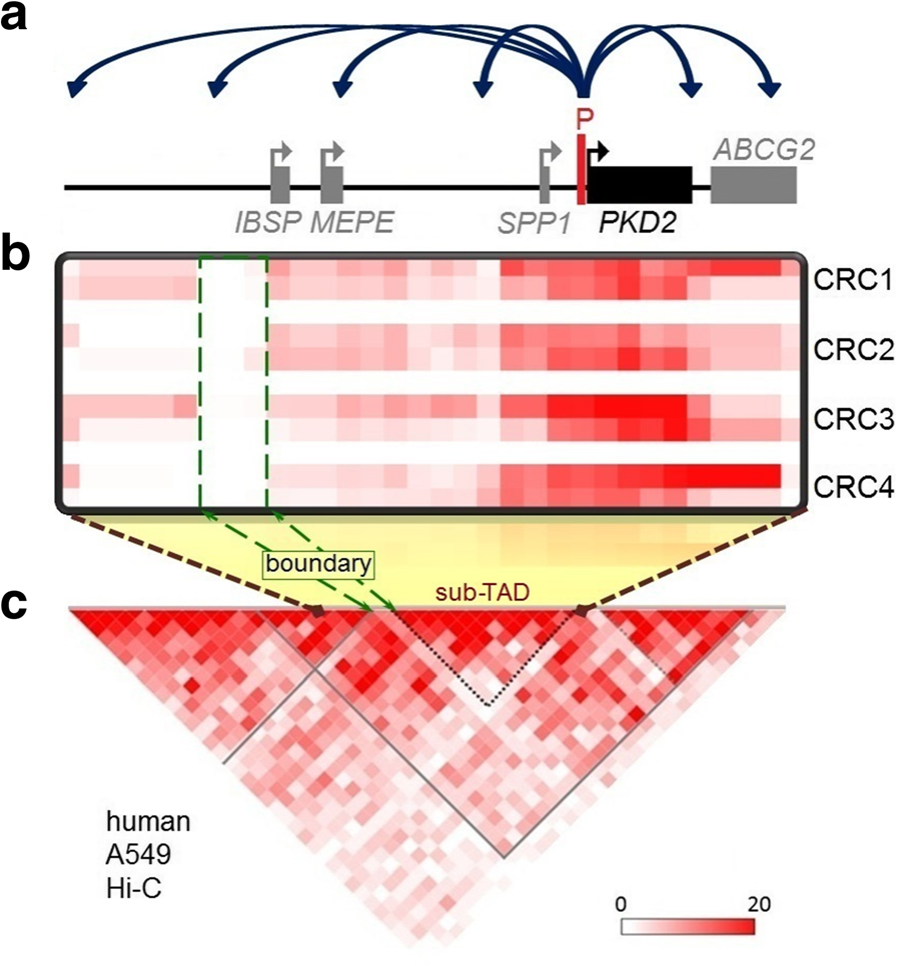
*PKD2* promoter is engaged in long-distance interactions. **a** Schematic linear representation of the locus under study. **b** 5C interaction maps of the *PKD2* promoter in four renal cyst epithelial cell (CRC) samples. Interaction frequencies were measured from the *PKD2* promoter region using two Reverse 5C primer “baits”. The 5C data from the two anchor points was averaged, binned at 80 kb (6× step) and are represented in heatmap form where interaction frequencies are colour-coded according to the respective scales. The average interaction frequencies are from the number of sequence reads after normalization. The heatmaps (two red lines for the two Reverse 5C primers) are aligned with the locus diagram shown in (**a**), and sample names are indicated on the right. **c** The regions interacting most frequently with the *PKD2* promoter are contained within *PKD2* sub-TAD. The A549 Hi-C data are shown in heatmap form as in (**b**)

We mapped the interaction profile of the *PKD2* promoter within the ~ 540 kb domain with 5C-seq in four CRC samples isolated from different individuals (Fig. 2a, b). We first observed that the chromatin environment of the *PKD2* promoter captured from either bait is generally similar in all samples (Fig. 2b). Variability is not unexpected in these types of analyses. Chromatin organization is notorious for its plasticity even within cell line samples. This variability has many sources including the different cell cycle phases of a culture sample, and whether or not the cells are exponentially growing and have a high transcription activity, and may come from genetic variations amongst the donors [60]. Interactions were preferentially found within the *PKD2* sub-TAD (Fig. 2b, c; dotted triangle). The highest levels of contacts were observed mostly within the *PKD2* gene and with the upstream and downstream regions containing *SPP1* and *ABCG2* genes, respectively. Importantly, we also detected long-range contacts between the *PKD2* promoter and multiple distal chromatin regions, particularly with one located 5′ of the *IBSP* and *MEPE* genes (Fig. 2b). As expected, we observed a reduction in the interaction frequency (highlighted in dashed green box), which corresponds to a TAD boundary (Fig. 2b, c). In fact, topological domains are separated by tight regions, *boundary regions*, where chromatin interactions are less frequent [48]. Altogether, these results suggest that the *PKD2* promoter engages in long-range contacts with several putative regulatory elements, which could be important in regulating its expression.

### *PKD2* contact regions display characteristics of regulatory elements

Some of the 5′ and 3′ regions engaged in long-range contacts with the *PKD2* promoter might correspond to DNA elements important for its regulation. We therefore tested if some of these regions displayed features of enhancers by using available databases for different markers of regulatory elements. We aligned our 5C interaction profiles from the four CRC samples with datasets from the human Renal Proximal Tubule Epithelial Cells (RPTECs). We assumed that the regulatory elements controlling *PKD2* in this cell line should be, at least in part, similar to the regulatory network in cyst epithelial cells. We specifically looked for the presence of DHS or H3K27Ac marks because these often reflect the presence of regulatory elements.

**Fig. 3:**
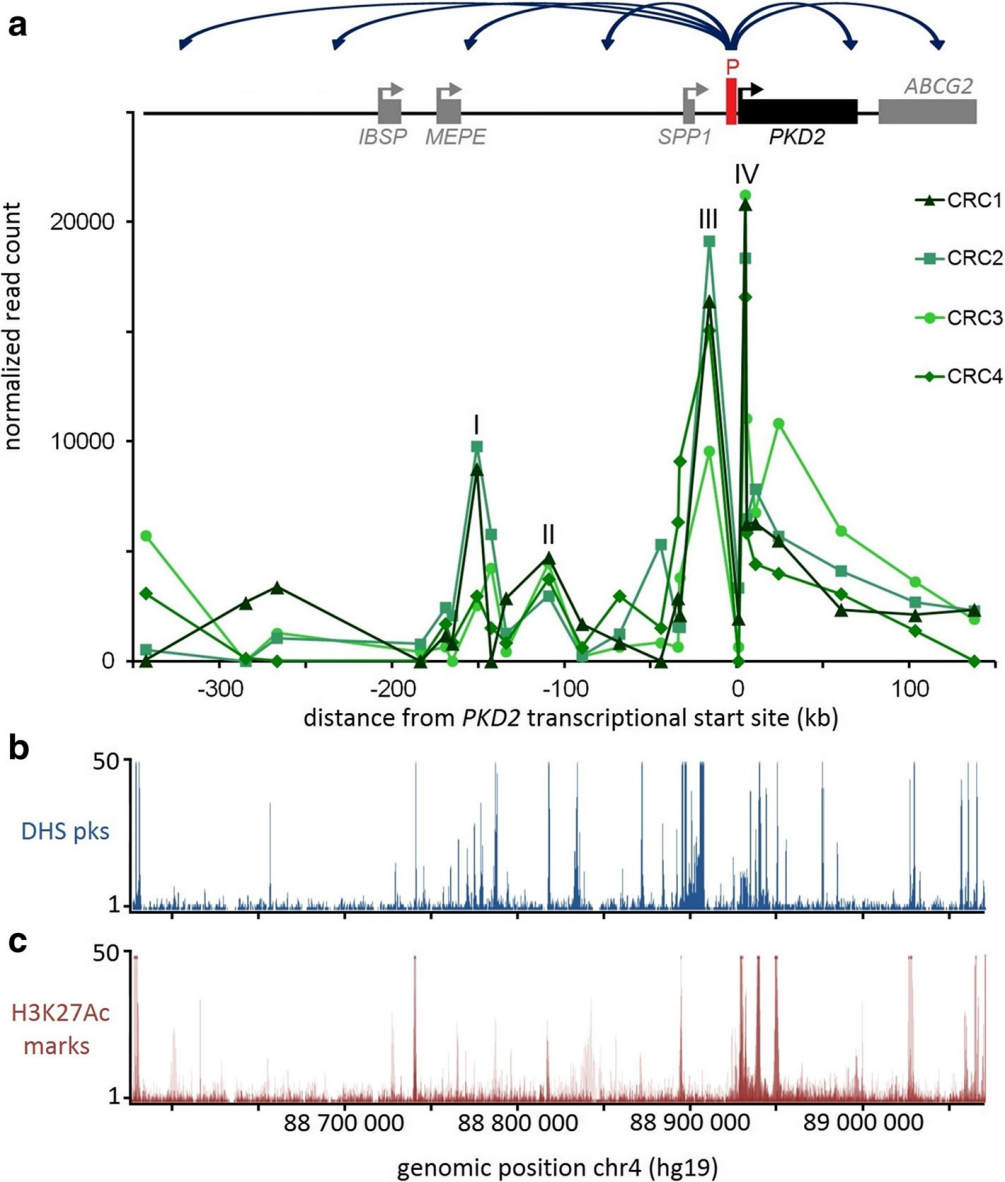
*PKD2* interacting regions display characteristics of regulatory elements. **a** 5C chromatin interaction profiles of the *PKD2* promoter region in four CRC samples. Interaction frequency (*y-axis*) is correlated with the position from the transcriptional start site of *PKD2* (*x-axis*). The four strong contact regions are numerated I to IV. **b** Alignment of DNAse I hypersensitivity data from RPTEC over the region characterized. **c** Alignment of H3K27Ac mark data from RPTEC over the region characterized

We found four strong chromatin interactions between *PKD2* promoter and the rest of the locus of interest in the four CRC samples (Fig. 3a, “I to IV”). These regions coincide with DHS and/or H3K27Ac marks (acetylation of the 27th lysine residue on histone protein 3) in RPTECs (Fig. 3b, c). Three contacts involve sequences within the 5′ region (Fig. 3a, ‘I to III’) and one is within the *PKD2* gene body (Fig. 3a, ‘IV’). These interacting regions were located between − 151 kb (for region I) and + 5 kb (for region IV) from the *PKD2* transcriptional start site (TSS).

### Several DHS regions show enhancer activities

**Fig. 4:**
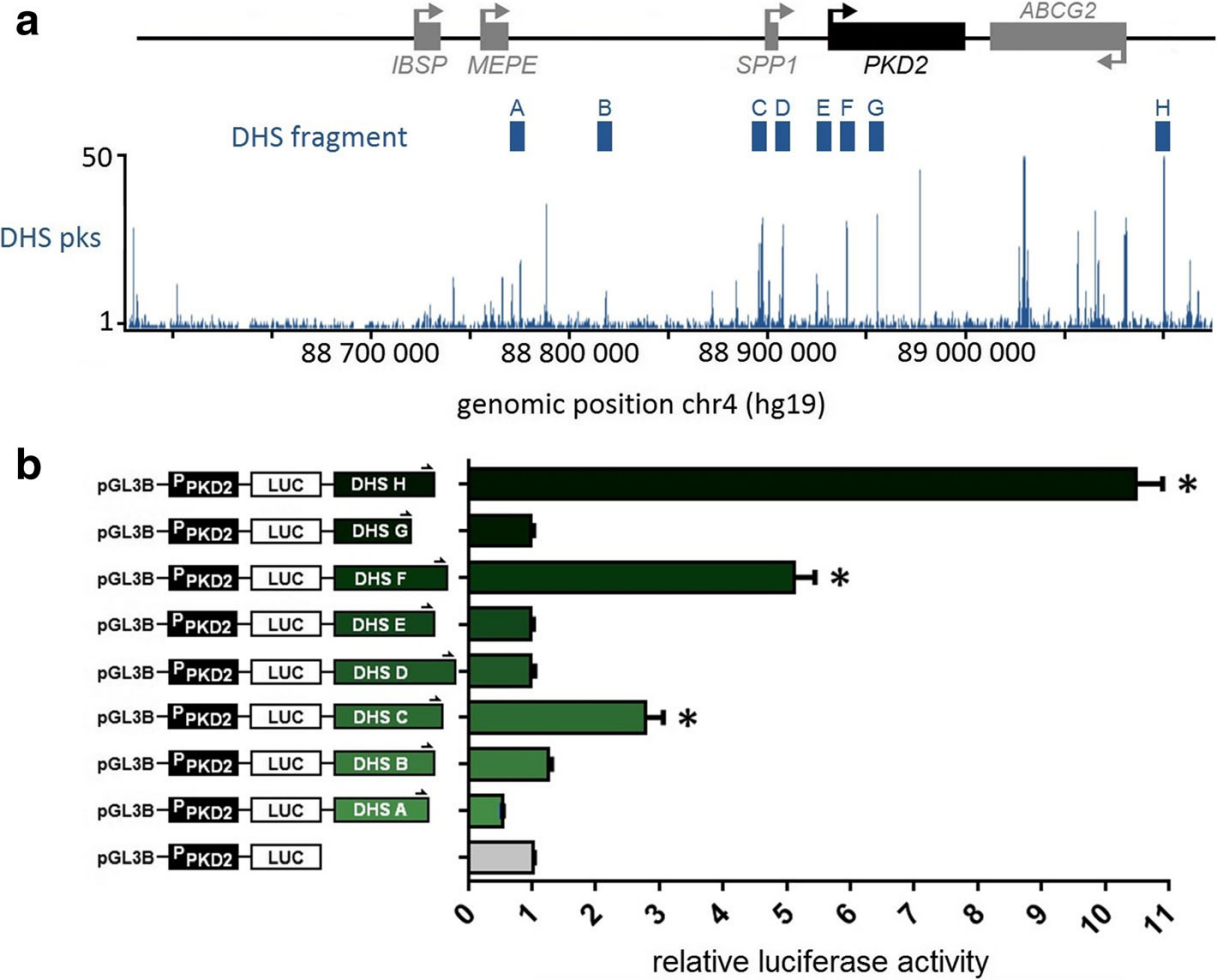
Several DHS regions show enhancer activities. **a** Alignment of DNAse I hypersensitivity data from A549 cells over the schematic linear representation of the region. Gene orientation is indicated with arrows. The cloned DHS elements are indicated with blue boxes at the top and identify open chromatin sites. **b** A549 cells were transfected with pGL3B luciferase reporter constructs containing the *PKD2* basal promoter (P_PKD2_; 1169 bp) and fragments of DHS elements of the region A to H. Luciferase data are shown relative to the *PKD2* basal promoter vector (=1). Error bars represent SEM (*n* = 9), * *P* < 0.0001 using unpaired t-tests

A common attribute of functional elements is their hypersensitivity to DNAse I, which corresponds to nucleosome-free regions where regulatory factors are bound. To further assess whether the region selected around *PKD2* for this study contains bona fide regulatory elements, we aligned available DHS data from A549 cells along our region and chose to test eight DHS fragments for enhancer activities against the *PKD2* promoter in a reporter assay (Fig. 4a, ‘A to H’). For this analysis, we first prepared a ‘P_PKD2_’ construct by subcloning a 1169 bp *PKD2* promoter fragment into the pGL3-Basic vector upstream of a modified firefly luciferase coding region optimized for analysing transcriptional activity in eukaryotic cells. Regions A to H were then PCR amplified and inserted into P_PKD2_ (Additional file 2: Table S2). These different constructs were individually co-transfected into A549 cells with a beta-galactosidase plasmid as a control for transfection efficiency. Firefly luciferase expression was measured after 48 h and was normalized against the *PKD2* promoter construct alone (P_PKD2_), which was set to 1.

As shown in Fig. 4b, three of our DHS fragments significantly enhanced *PKD2* promoter activity. The fragment encompassing DHS H (hg19, chr4:89,099,146-89,100,467), located at + 170 kb from *PKD2* TSS, displayed a strong enhancer function with more than ten-fold effect on the *PKD2* promoter. The DHS region F (hg19, chr4: 88,938,944-88,940,420), located at + 10 kb from *PKD2* TSS, increased the promoter activity by 5.1-fold and DHS C (hg19, chr4:88,895,792-88,897,206), located at − 32 kb from *PKD2* TSS, had a more modest enhancer activity with an almost three-fold effect. However, we observed no evidence of cooperation among these three elements (data not shown). The other DHS regions did not show any enhancer activity in A549 cells.

### Formation of a *PKD2* “active chromatin hub”

**Fig. 5:**
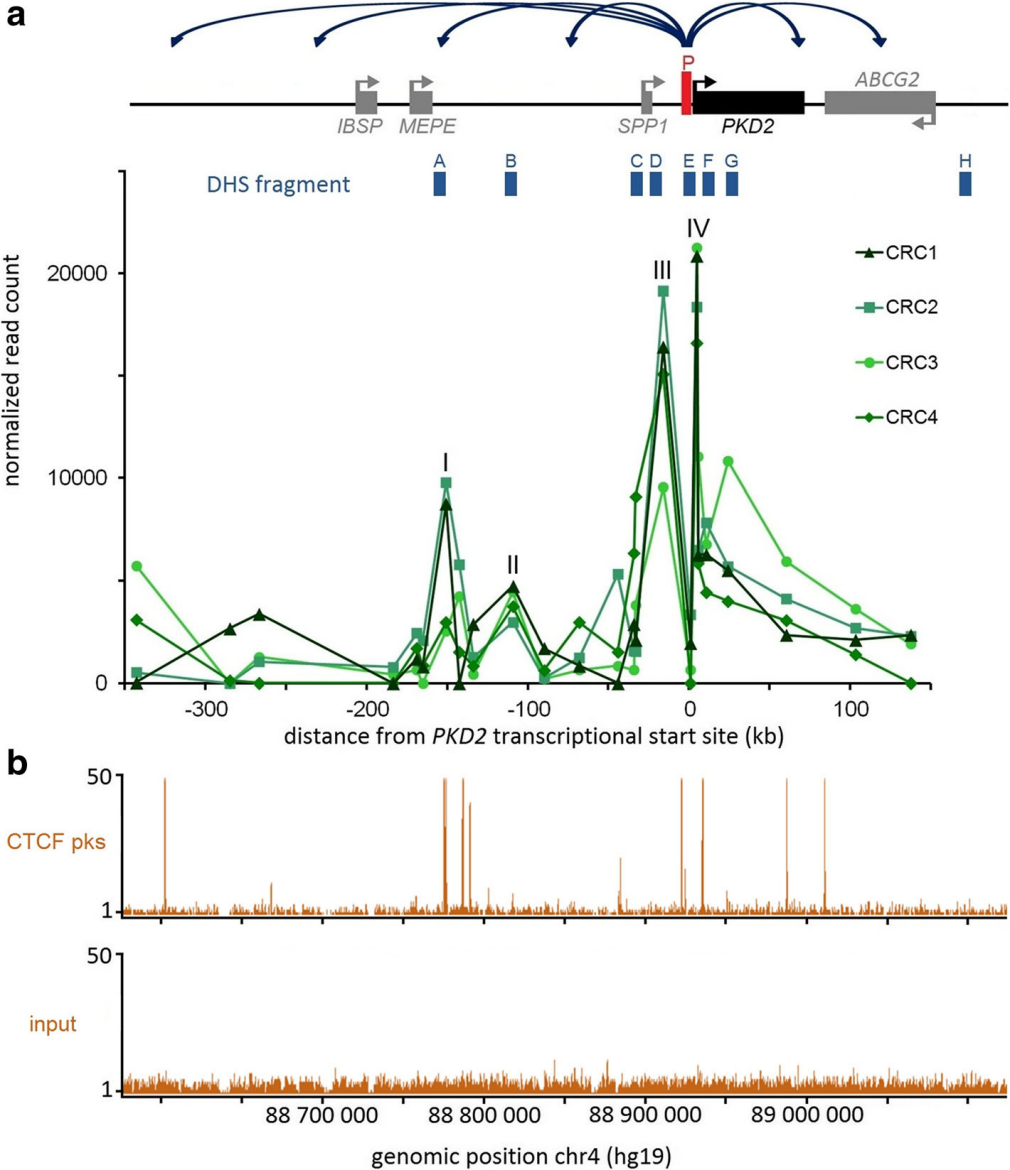
Formation of a *PKD2* “active chromatin hub”. **a** Alignment of 5C chromatin interaction profiles of the *PKD2* promoter region in four CRC samples with the cloned DHS elements to highlight the position of enhancers C, F and H relative to interacting regions. **b** Alignment of CTCF binding sites from RPTEC to highlight the position of enhancers and interacting regions relative to CTCF peaks

The four regions (Fig. 5a, ‘I to IV’) that we found to strongly interact with the *PKD2* promoter in four CRC samples with 5C analysis, correspond to DHS fragments A, B, D and E, respectively, and do not show *PKD2* enhancer activity. However, some of these regions are localized at more or less close proximity to strong CTCF peaks in RPTECs (Fig. 5b). CTCF functions as a genome architectural protein organizer that contributes to create local chromatin hubs, to join clusters of genes with coordinated expression and to promote communication between regulatory elements and their corresponding promoters [61]. A Subset of chromatin loops that are formed by a CTCF-CTCF homodimer brings sequences that are far apart in the linear genome into close proximity [62]. Thus, CTCF-associated interactions potentially allow extensive cross-talk between promoters and distant enhancers [63]. CTCF is sometimes referred to as an enhancer facilitator [61]. CTCF binding is largely invariant across cell types in mammals [64], and so these nucleosome-free regions could be implicated in the formation of a *PKD2* ‘active chromatin hub’ due to the recruitment of CTCF factors. Moreover, enhancers C and F are located directly near DHS fragments D and E, which are respectively not so far and at close proximity of CTCF binding sites. Thus, we suggest that these enhancers activate the *PKD2* promoter by achieving physical proximity via CTCF-CTCF binding and looping of the intervening chromatin.

## Discussion

The crucial step in the development of cysts in ADPKD is linked to the amount of functional PC1 or PC2, and a reduction in the amount of these functional polycystins (also called the dosage model) leads to cystogenesis [65]. It is possible that the threshold could vary between tissues and cell types as well as with the type of mutated allele. This highlights the importance of understanding the regulation and expression of polycystins in order to discover new targets to increase their expression and possibly decrease cyst formation and slow disease progression.

Because the regulatory mechanisms of *PKD2* transcription remain largely uncharacterized and some cases of ADPKD have no genotypic explanation [7], it is essential to investigate alternative control mechanisms, particularly long-range regulation. We used 5C technology to identify regions that interact specifically with the *PKD2* promoter and may be involved in regulating its expression. We decided to study these interactions in primary cells to avoid altered expression patterns of cell lines. For this purpose, we used human renal epithelial tissues obtained from nephrectomy.

Despite some minor variations among cell samples, our results show that the *PKD2* promoter is engaged in numerous long-range interactions (Fig. 2a). These chromatin contacts are mostly located within the *PKD2* sub-TAD (Fig. 2b, c). These interacting partners lie downstream and upstream of extragenic regions and within intronic regions, preferentially within the *PKD2* gene as well as just upstream and downstream of regions containing *SPP1* and *ABCG2* genes. Interactions of the *PKD2* promoter with neighbouring regions decrease substantially with distance, especially at a region corresponding to a TAD boundary (dashed green box, Fig. 2b, c). This is consistent with studies that recently showed that chromatin interactions within *boundary regions* is practically non-existent [48, 52].

Our 5C data revealed four strong interacting regions that correlate with marks of regulatory elements (Fig. 3a-c). For example, we detected contacts between the *PKD2* promoter and a region located ~ 150 kb upstream (Fig. 3a, region I) containing DHS sites (Fig. 3b) and a high CTCF binding site (Fig. 5a, b).

We also found interactions between the promoter and an element located at ~ 110 kb from the *PKD2* TSS in 5′ region (region II), which coincides with DHS and H3K27Ac marks. Furthermore, we detected interactions with two others regions located at 16 kb upstream and 5 kb downstream of the *PKD2* TSS (region III and IV), which correspond to strong DHS, H3K27Ac marks and are at proximity of CTCF binding sites.

In addition, by using reporter assays where luciferase expression is driven by a *PKD2* promoter fragment, we found that three DHS regions act as enhancers (Fig. 4b). The region H, located at + 170 kb of the *PKD2* promoter show the strongest enhancer activity with more than 10-fold increase of the *PKD2* expression in A549 cells. DHS C at approximately − 32 kb and DHS F at approximately + 10 kb from the *PKD2* TSS have more modest enhancer activities, increasing the promoter activity by 2.8- and 5.1-fold, respectively. These three regions span sequences found to bind different types of transcription factors and chromatin remodelers in an ENCODE compilation of 161 factors with Factorbook motifs in 91 cell types (Additional file 3: Figure S1). To identify the transcription factors driving enhancer activities, these DHS regions were evaluated in subfragments. These segments showed a more modest activity even no enhancement compared with entire regions (data not shown). These results suggest that combinatorial regulatory elements would be involved [66, 67]. For example, transcription factors FOS and JUND could act cooperatively in region F.

In addition, others regions (Fig. 3, regions I to IV that correspond to DHS A, B, D and E, respectively) that correlate with regulatory markers and interact with the *PKD2* promoter without increasing its expression, may still function in the regulation of *PKD2* through other mechanisms or by acting in a combinatorial fashion. In particular, enhancers C and F are located directly near DHS fragments D and E which are respectively not so far and at close proximity of CTCF binding sites, suggesting that the increase of the *PKD2* promoter by these *cis*-acting regulatory elements could depend on the active chromatin looping driven by these insulator factors which bring them into spatial proximity.

In conclusion, we identified a new set of *PKD2* distal *cis*-regulatory elements in primary human renal epithelial cells. We proposed a looping model in the organization of the *PKD2* locus in renal cells via the formation of a chromatin loop with CTCF-CTCF binding around the gene.

It is evident that other sequence variabilities in the coding region, in the promoter sequence or at a distance from the promoter region of the *PKD* genes are important to explain the regulation of the promoter activity, which can influence cyst formation [68].

Future work will be aimed at identifying this long-range regulation of *PKD2* expression and, in particular, the trans-acting factors that mediate it in greater detail.

Identification of long-range acting regulatory elements that affect expression of genes may also be important for genetic diagnosis of ADPKD. It will also be very interesting to study *cis*-regulation with the *PKD1* promoter and potential co-regulation between these two genes. Mutations in these regulatory elements can also explain many cases of patients who have either a hypomorphic allele in the *PKD1* or *PKD2* gene coding sequences or none at all. Moreover, genetic alterations within remote-acting regions could lead to *PKD* dysregulation, which might explain variability in phenotypic features of ADPKD, such as the development of renal insufficiency [69]. Some regulatory variants have already been associated to extensive phenotypic variability. Indeed, recently non-coding variants have been related with cystic fibrosis disease traits [70]. Thus, these *PKD2 cis*-regulatory elements could be sequenced in ADPKD patients in whom no mutation was found in the *PKD* gene coding sequences or patients with extreme clinical phenotypes. Maybe, with time, these regions would be analysed by whole-genome sequencing [71].

## Conclusion

In conclusion, we reported for the first time the use of 5C-seq technology in primary human cells to analyse the chromatin conformation of a locus involved in ADPKD and identified new regulatory elements. We demonstrated that three regions surrounding the *PKD2* gene interact with the promoter and activate its expression in renal epithelial cells. We proposed a regulatory looping model in the 3D-organization of the *PKD2* locus in renal cells via the formation of a chromatin loop thanks to the recruitment of CTCF.

These findings provide valuable information on the basic insights of the regulation of this gene, and the description of *PKD2* regulatory elements may be important for gene targeting constructs for therapy development. They also afford new targets for mutation screening in genetic diagnosis of ADPKD.

## Methods

### Cell collection and culture

Human renal tissues were obtained from PKD1 patients with ADPKD (carrying no *PKD2* mutation) from the Genkyst cohort who had undergone nephrectomy. Several cyst tops were individually dissected with a scalpel. Each cyst was rinsed thoroughly in ice-cold phosphate buffered saline (PBS) and then placed in a separate tube containing 0.15% Pronase E. Cysts were incubated for 24 h at 4 °C to allow separation of cells from the cyst wall. Dissociation was stopped by addition of foetal bovine serum (FBS). The cyst walls stripped of cells were removed by filtration, and the cells were washed twice in PBS. Renal cyst epithelial cell (CRC) suspensions were centrifuged for 5 min at 2200 *g* at 4 °C. Cell pellets were resuspended in a selected and optimized cell culture media for human renal epithelial cells (REGM; Lonza), and cells were cultured following the manufacturer’s instructions.

A549 human lung carcinoma cells [72] were grown in Dulbecco’s modified eagle medium (DMEM; Lonza) with 10% FBS.

All cells were grown on plastic at liquid interface at 37 °C in 5% CO_2_ saturated humid air.

### Cell isolation and fixation for 5C analysis

CRC cells were fixed with 1.5% formaldehyde for 10 min at room temperature. Crosslinking was stopped with glycine (125 mM final concentration) by incubating 5 min at room temperature followed with 15 min on ice. Cells were scraped and centrifuged at 400 *g* for 10 min at 4 °C. Supernatants were removed and the cell pellets were quick-frozen on dry ice.

### 3C library preparation

The fixed CRC cell pellets were treated as previously described [73, 74]. Briefly, 10 million cells were incubated in lysis buffer (10 mM Tris (pH 8.0), 10 mM NaCl, 0.2% NP-40, supplemented with fresh protease inhibitor cocktail) for 10 min on ice. Cells were then disrupted on ice with a Dounce homogenizer (pestle B; 2 × 20 strokes). Nuclei were recovered by centrifugation, washed twice with 1X EcoRI buffer (NEB) and resuspended in 100 μl of 1X EcoRI buffer.1X EcoRI buffer (337 μl) was added to 50 μl of cell suspension, and the mixture was incubated for 10 min at 65 °C with 0.1% SDS final concentration (38 μl). Triton X-100 (44 μl of 10% Triton X-100) was added before overnight digestion with EcoRI (400 U). The restriction enzyme was inactivated on the next day by adding 86 μl of 10% SDS and incubating 30 min at 65 °C. Samples were then individually diluted into 7.62 ml of ligation mix (750 μl of 10% Triton X-100, 750 μl of 10X ligation buffer, 80 μl of 10 mg/ml BSA, 80 μl of 100 mM ATP and 3000 Cohesive end Units of T4 DNA ligase). Ligation was carried out at 16 °C for 2 h.

The 3C libraries were next incubated overnight with 45 μL of Proteinase K (10 mg/ml) at 55 °C.The DNA was purified by phenol-chloroform extraction and precipitated with 3 M NaOAc pH 5.2 (800 μl) and cold ethanol. After at least 1 h at − 80 °C, the DNA was recovered by centrifugation, and the pellets were washed with cold 70% ethanol and then resuspended in 400 μl of 1X TE pH 8.0. A second phenol-chloroform extraction and precipitation with 3 M NaOAc pH 5.2 (40 μl) and cold ethanol was performed. DNA was recovered by centrifugation and washed eight times with cold 70% ethanol. The pellets were finally dissolved in 100 μl of 1X TE pH 8.0 and incubated with RNAse A (1 μl at 10 mg/ml) for 15 min at 37 °C.

### 5C primer and library design

5C primers covering the *PKD2* region (hg19, chr4: 88,560,000-89,230,000), the *ERCC3* region (hg19, chr2:128,014,866-128,051,752) and the *ENr313* region (hg19, chr16:62,276,449-62,776,448) were designed using “my5C.primer” [75] with the following parameters: optimal primer length of 30 nt, optimal TM of 60 °C, default primer quality parameters (mer: 800, U-blast: 3, S-blasr: 50). Primers were not designed for large (> 20 kb) restriction fragments. Low complexity and repetitive sequences were excluded from our experimental design such that not all fragments could be probed in our assays. Primers with several genomic targets were also removed.

The universal A-key (CCATCTCATCCCTGCGTGTCTCCGACTCAG-(5C-specific)) and the P1-key tails ((5C-specific)-ATCACCGACTGCCCATAGAGAGG) were added to the Forward and Reverse 5C primers, respectively. Reverse 5C primers were phosphorylated at their 5′ ends. An anchored 5C design was used for the *PKD2* locus, 2 Reverse 5C primers targeted the *PKD2* promoter while 24 Forward 5C primers covered the surrounding region.

For the control regions, an alternating 5C design was used: alternating Forward and Reverse 5C primers covering *ERCC3* and *ENr313* regions were used to generate the 5C libraries. This design used 31 primers (2 Forward / 3 Reverse for the *ERCC3* region, 13 Forward / 13 Reverse *ENr313* region). All 5C primer sequences are listed in Additional file 1: Table S1.

### 5C library preparation

5C libraries were prepared and amplified with the A-key and P1-key primers following a procedure described previously [57]. Briefly, 3C libraries were first titrated by PCR for quality control (single band, absence of primer dimers, etc.) and to verify that contacts are amplified at frequencies similar to what is usually obtained from comparable libraries (same DNA amount from the same species and karyotype) [73, 76, 77].

The 5C primer stocks (20 μM) were diluted individually in water on ice, and mixed to a final concentration of 0.002 μM. The mixed primers were combined with annealing buffer (10X NEBuffer 4, New England Biolabs Inc.) on ice in reaction tubes. Salmon testis DNA was added to each 5C reaction, followed by the 3C libraries and water for a final volume of 10 μl. Samples were denatured at 95 °C for 5 min, and annealed at 50 °C for 16 h before ligation with Taq DNA ligase (10 U) for one hour. Each ligation reaction was then PCR-amplified individually with primers against the A-key and P1-key primer tails. We used 35 cycles based on dilution series showing linear PCR amplification within that cycle range. The 5C PCR products of corresponding 3C libraries were pooled before purifying the DNA on MinElute columns (Qiagen).

The 5C libraries were quantified on agarose gel and diluted to 0.012 ng/μl (for Ion PGM™ Template OT2 200 Kit). A 25 μL volume of the diluted 5C library was used for sequencing with an Ion PGM™ sequencer. Samples were sequenced onto Ion 314™ Chips v2 or Ion 316™ Chips following the Ion PGM™ Template OT2 200 Kit and Ion PGM™ Sequencing 200 Kit v2 protocols recommended by the manufacturer (Life Technologies™).

### 5C data analyses

Analysis of the 5C sequencing data were performed as described previously [57, 59]. The sequencing data were processed through a Torrent 5C data transformation pipeline on our local instance of Galaxy as previously described [57, 78] (http://galaxy.bci.mcgill.ca/galaxy/). This analysis generates an excel sheet containing interaction frequency lists (IFL) as well as a text file, which were used to visualize results using “my5C-heatmap” [75].

Normalization between different libraries was done first by read count and then by using the compaction profiles for the *ERCC3* region and the gene desert region *ENr313* after setting one sample (CRC) as a reference.

The *ERCC3* and *ENr313* 5C data for each sample were normalized by dividing the number of reads of each 5C contact by the total number of reads from the corresponding sequence run. A ratio, calculated by dividing these normalized data by normalized data from a reference sample (CRC), was applied to the corresponding raw data of the study region for each sample.

5C data was averaged, optimally binned at 80 kb (6× step) and are represented in heatmap form where interaction frequencies are colour-coded according to the respective scales. The average interaction frequencies are from the number of sequence reads after normalization. Data was binned based on distance for several reasons. First, by binning the data and re-dividing it into defined genomic distances (or “steps”), 5C signals are averaged along the region such that 5C contacts with greater error rates will not be seen as false positive or negative. This is particularly important given that the data is displayed on a color scale and that the eye does not detect signal linearly. By binning and re-dividing we also avoid displaying gaps which my5C inserts as grey lines that detracts attention from the actual findings. At the same time, this transformation allows us to align the data directly onto the genomic region without having to use “true size” - a display of the data where bin sizes are the same as the fragments sizes and which can enhance or reduce contact frequency to the eye. “True size” displays also insert grey lines in the heatmaps.

### Databases and URLs

The Hi-C data from human A549 were downloaded from computational and functional Genomics/Epigenomics Yue Lab website at http://promoter.bx.psu.edu/hi-c/view.php. The Hi-C data shown in a heatmap format were binned at 40 kb. TAD and sub-TADs were outlined manually based on clustering of interaction frequencies.

DHS peaks, CTCF binding sites datasets of H3K27Ac marks for RPTEC and A549 and transcription factor ChIP-seq data of 161 factors from 91 cell types were obtained and visualized using the UCSC genome browser (http://genome.ucsc.edu) [79].

The “my5C-primer” and “my5C-heatmap” bioinformatics tools are found at http://3dg.umassmed.edu/my5Cheatmap/heatmap.php.

### Plasmid constructs

All the cloning steps were done using the “In-fusion®” strategy from Clontech. Using the pGL3-Basic Vector (Promega), the 5′-flanking region of the *PKD2* gene (1169 bp, “P_PKD2_”) was cloned upstream of the firefly luciferase cDNA at the Hind III site. The candidate enhancer elements (A to H) were amplified and inserted downstream of the P_PKD2_ construct.

All the inserted fragments were verified by sequencing. The PCR primers used to amplify the *PKD2* promoter and candidate enhancer sequences are shown in Additional file 2: Table S2.

### Luciferase assay

Cells (2.5 × 10^5^) were seeded in 6-well plates. Transfections were done 24 h later with the transit 2020 reagent (Mirus). 800 ng of the P_PKD2_ construct and 200 ng of the pCMV-LacZ construct (as internal control) were used for each condition. Every condition was carried out in triplicates.

The cells were washed once with 1X PBS and lysed with Passive lysis buffer (Promega) 48 h post-transfection. Cell lysates were clarified by centrifugation at 15,000 *g* for 5 min at 4 °C. 20 μL of each protein extract was used to assay the luciferase activity and 25 μL for beta-galactosidase activity. We used Promega reagents and a multiwell plate reader Varioskan (Thermo Fisher). The results were presented as relative luciferase activity with the P_PKD2_ construct activity equal to 1. Significance determination of the increased luciferase activity was performed using unpaired *t*-tests using the GraphPad Prism® software.

## Additional files

Additional file 1: **Table S1.** 5C primer sequences for the *PKD2*, *ERCC3* and ENr313 regions. (PDF 75 kb)

Additional file 2: **Table S2.** PCR primer sequences used for cloning into the luciferase reporter construct (5′ - 3′). (PDF 12 kb)

Additional file 3: **Figure S1.** Enhancer regions overlap with regulatory element binding sites. Enhancer regions C (A), F (B) and G (C) overlap with several transcription factor or chromatin remodeler binding sites with Factorbook motifs identified by ChIP-seq in 91 different cell lines. (TIF 385 kb)

## Abbreviations

3C: Chromosome conformation capture
5C: Chromosome conformation capture carbon copy
5C-seq: Chromosome conformation capture carbon copy - sequencing
ADPKD: Autosomal dominant polycystic kidney disease
CRC: Renal cyst epithelial cells
CTCF: CCCTC-binding factor
DHS: DNAse I hypersensitive sites
PC: Polycystin
PKD: Polycystic kidney disease
RPTECs: Renal proximal tubule epithelial cells
TAD: Topologically associated domain
TSS: Transcriptional start site
