# Supplementary material for: Novel long-range regulatory mechanisms controlling PKD2 gene expression
Source: BMC Genomics. 2018 Jul 3;19:515. doi: 10.1186/s12864-018-4892-6 (PMC6038307; doi:10.1186/s12864-018-4892-6)
Supplement: Supplementary file 1 — Table S1. 5C primer sequences for the PKD2, ERCC3 and ENr313 regions. (PDF 75 kb) [file 12864_2018_4892_MOESM1_ESM.pdf]

Table S1. 5C primer sequences for the *PKD2, ERCC3* and *ENr313* regions

Fragment	Type	Size (nt)	Genomic sequence (5' to 3')	EcoRI position start	EcoRI position end
***PKD2 locus* (chr4; hg19)**					
1	Forward	28	CTCCCACTTCCACTGCAGTACCTCAGAA	88580796	88585969
2	Forward	30	GCTTGAGAAATTTATAATGAGACTTGGGAA	88639527	88643768
3	Forward	30	ACAAATGATTAGTAGTGAGCATTGACTGAA	88650767	88662223
4	Forward	30	AAGATAAGACCACCATTAATATGTACAGAA	88740973	88745239
5	Forward	29	CTGTGTGGAAGAGCAGAGGGTAAACAGAA	88755141	88759847
6	Forward	30	CTGATTTGGAACCCCACAAACAAAAGGGAA	88760732	88763729
7	Forward	30	TGAGAGAATCTGATGCATCCATTAGCTGAA	88769632	88778091
8	Forward	30	ACTACTCCAGTCATGTTGATACCATCTGAA	88784994	88786234
9	Forward	30	GGAAACTTGTTGGTAAAAATCTTGAATGAA	88792940	88794899
10	Forward	28	CGACAGAGCTACCAACATCCAAGGTGAA	88813978	88819680
11	Forward	30	AGAAAGAACAATATGGAATGCATTCCAGAA	88836797	88838865
12	Forward	30	TTTAGGTAGCAGTTTCCATAAAAAGCAGAA	88859401	88860788
13	Forward	30	TTTACTCCAACACTTAGCTTTCTTTTAGAA	88878075	88884703
14	Forward	29	GCATCCCAGACACATTCTTTACCCGTGAA	88893530	88894598
15	Forward	25	GACCCAGAGCTCTGCTATCCCTGAA	88894599	88895675
16	Forward	30	ATACAGATCAGCTCACAAACATCAACTGAA	88905986	88912731
19	Forward	24	CTATTTCCGGTACCCAGCGCGGAA	88925002	88929662
20	Forward	30	ATCATACCAACACAGTCCGTGTATCCAGAA	88929663	88933651
21	Forward	30	GGTTGTGGTAAGGTTGTGCCAGTAACAGAA	88933652	88934398
22	Forward	30	ACGTATGATGCGTAACACAGTGTTTTTGAA	88938271	88939367
23	Forward	30	TTTGATGTTCTGTTATTCAATACATAAGAA	88945197	88952989
24	Forward	28	GACCAGGCAGTTCCCTCATCTCTCTGAA	88983649	88989290
25	Forward	24	CCACTGCTCTGAACATGGCTGGAA	89027829	89032122
26	Forward	30	GTAGAAGCCAAGATAGCCTCAAGTTGAGAA	89063894	89066226
R1 (17)	Reverse	30	TTCTGCCAGCTCACTTTTCATTTTTCCATG	88918554	88918906
R2 (18)	Reverse	30	TTCCTTCCTTTAATACCCATCTGAAGACCT	88920631	88924619
***ERCC3* (chr2; hg19)**					
27	Forward	25	ATGAATATGCCGCACAAAGCAAGAA	128025461	128026627
28	Forward	20	ACAGCAAACGGGGCTGAGAA	128031121	128045371
29	Reverse	22	TTCCATATGGGGCAGTTGTTCC	128026628	128029466
30	Reverse	25	TTCATGTCTGCTGAAGGAACTTCTC	128029467	128030358
31	Reverse	23	TTCAGTATGGTACTGCAGCCAGT	128045372	128051752
***Enr313* (chr16; hg19)**					
1	Forward	30	AATATTTATGGGCAATTCCCATAAAGGGAA	62276449	62288079
2	Forward	30	ACCACCACAACCTTAATAAAGTGAATAGAA	62322529	62322909
3	Forward	29	CAAAGAGCTGAAACAATTGTCTTCCAGAA	62350920	62351186
4	Forward	28	GACAGAGTTTGCCTTTTCTGTAGAAGAA	62396323	62397973
5	Forward	30	CTTTAGGCTTGTACTAGCAAAATGTAAGAA	62430033	62436835
6	Forward	20	GCAGGCCCTGGTGGGGTGAA	62505188	62510752
7	Forward	30	ACTCTTCAATGATAAGAATTGATGCCTGAA	62560843	62566296
8	Forward	30	ACATACTTCAAATTATACCGACTCTTGGAA	62570729	62571192
9	Forward	25	GAGATGTTGATGGCCATATGCTGAA	62630894	62632252
10	Forward	28	TCTCAAGTCCTGATTTTGTCATCCTGAA	62657702	62658396
11	Forward	30	GAATGTTTAAAGTTTGATGCATTCATAGAA	62687940	62690686
12	Forward	30	TTATAAATGACTTCACAAATGACTTTAGAA	62733577	62734821
13	Forward	28	GATGTCACTCAGTAAGCTTGAGTTTGAA	62761022	62762511
14	Reverse	30	TTCTAAATAGCTGAGGTTATAGTGACCATA	62294294	62298437
15	Reverse	30	TTCCTACTATTTGTCAGATTGCTAGGTAAT	62320192	62322528
16	Reverse	30	TTCTAGTTCAGGAGAATAAAAGTTATATCA	62337062	62337208
17	Reverse	30	TTCACATCCATTCACATGATGTTTCTAAAT	62351306	62360362
18	Reverse	30	TTCAGGTAATCAAAAACATCAGTAATGTTT	62383166	62386184
19	Reverse	30	TTCTGTACCATATTCAGCTAGAAATTTTCT	62436836	62442745
20	Reverse	30	TTCTGAGGAATAGAGAGCTAAGAAACTCAC	62496014	62498840
21	Reverse	30	TTCTCAAAGTGGTTTTCTATTGGATTATAT	62534799	62537279
22	Reverse	30	TTCTATCTTCCTTAAGAAAAAATTCTGTCT	62566297	62566973
23	Reverse	30	TTCCCTTCATTCAATGGAATAATTGCAGAG	62630674	62630893
24	Reverse	30	TTCAGTATAAATCCCTCAGTGCATTTATCA	62677376	62687939
25	Reverse	30	TTCTATGCTATTAACTAAATTATTGAGTTT	62734822	62736992
26	Reverse	30	TTCTAGTGATAGATTACAGCTGAGACAAGA	62743987	62761021
