# Supplementary material for: Novel long-range regulatory mechanisms controlling PKD2 gene expression
Source: BMC Genomics. 2018 Jul 3;19:515. doi: 10.1186/s12864-018-4892-6 (PMC6038307; doi:10.1186/s12864-018-4892-6)
Supplement: Supplementary file 2 — Table S2. PCR primer sequences used for cloning into the luciferase reporter construct (5′ - 3′). (PDF 12 kb) [file 12864_2018_4892_MOESM2_ESM.pdf]

Table S2. PCR primer sequences used for cloning into the luciferase reporter construct (5' - 3')

			genomic position chr4 (hg19)		distance from the *PKD2* transcriptional start site (kb)	
P_{PKD2}	F	CGATCTAAGTAAGCTTCCACAGAGGTGTCTAAGCATC	88 927 663	88 928 831		
	R	CCGGAATGCCAAGCTTCTCAGGAGCCATGTTCCTTTC				
Element A	F	ATAAGGATCCGTCGACCCCTTCTAAAGTGGTGTCATAGG	88 777 321	88 778 349	-151,5	-150,5
	R	AAGGGCATCGGTCGACCAAGTCTCAGACCGAATGCTAC				
Element B	F	ATAAGGATCCGTCGACGGTAATCCAAGTAGCAGTTTACTC	88 816 818	88 818 195	-112,0	-110,6
	R	AAGGGCATCGGTCGACGTAGGTGCTACAGTCCAATGAC				
Element C	F	ATAAGGATCCGTCGACGCCTACTTTACATACCTTGGGAG	88 895 792	88 897 206	-33,0	-31,6
	R	AAGGGCATCGGTCGACGATTAGCATCGGTGGTTTCCG				
Element D	F	ATAAGGATCCGTCGACCCCACTGCTGAGCATATAACC	88 905 470	88 907 438	-23,4	-21,4
	R	AAGGGCATCGGTCGACCTTCCTCAATTATTTCCTTGTGGG				
Element E	F	ATAAGGATCCGTCGACCCAGGCACTTTGTGTTCAAAGTG	88 933 490	88 934 860	4,7	6,0
	R	AAGGGCATCGGTCGACGATACAGGCTCTCACCTCTCA				
Element F	F	ATAAGGATCCGTCGACGCTGTTCCTTAGTGCCTAGTTG	88 938 944	88 940 420	10,1	11,6
	R	AAGGGCATCGGTCGACCATCTGGCTCCAATGTTACCC				
Element G	F	ATAAGGATCCGTCGACCTGCCTATTCTCAGGCAGTTC	88 949 116	88 950 097	20,3	21,3
	R	AAGGGCATCGGTCGACGGTAAAGGCTGTTGACACAAGTG				
Element H	F	ATAAGGATCCGTCGACCCACGAAAACAGCAATTCCTGG	89 099 146	89 100 467	170,3	171,6
	R	AAGGGCATCGGTCGACCACAGAGGTAAAATCTGGTGGTC				
