# Supplementary figures and images for: Novel long-range regulatory mechanisms controlling PKD2 gene expression
Source: BMC Genomics. 2018 Jul 3;19:515. doi: 10.1186/s12864-018-4892-6 (PMC6038307; doi:10.1186/s12864-018-4892-6)

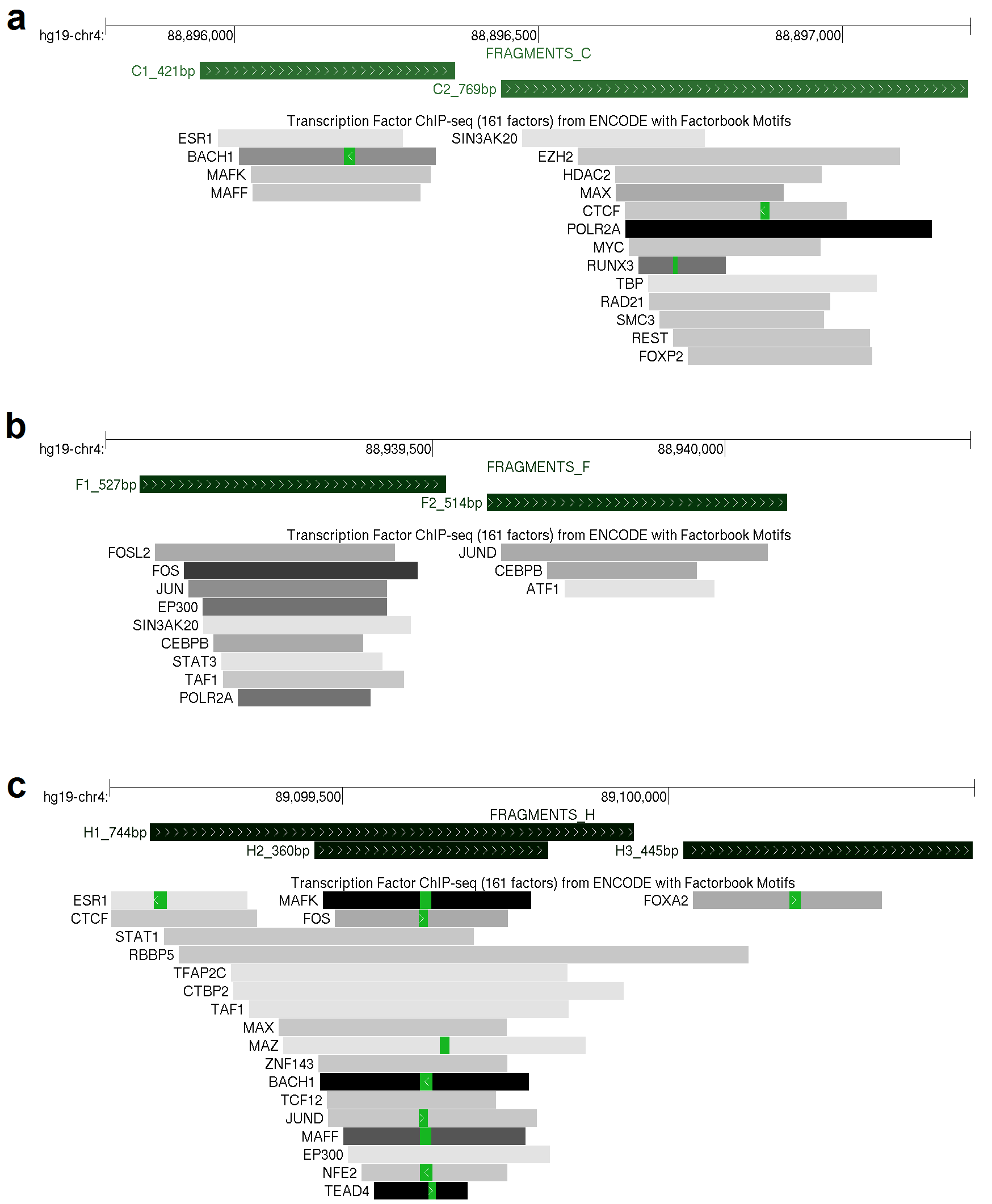

Supplement: Supplementary file 3 — Figure S1. Enhancer regions overlap with regulatory element binding sites. Enhancer regions C (A), F (B) and G (C) overlap with several transcription factor or chromatin remodeler binding sites with Factorbook motifs identified by ChIP-seq in 91 different cell lines. (TIF 385 kb) [file 12864_2018_4892_MOESM3_ESM.tif]
